# The multiplicity of malaria transmission: a review of entomological inoculation rate measurements and methods across sub-Saharan Africa

**DOI:** 10.1186/1475-2875-8-19

**Published:** 2009-01-23

**Authors:** Louise A Kelly-Hope, F Ellis McKenzie

**Affiliations:** 1 Vector Group, Liverpool School of Tropical Medicine, Liverpool, UK; 2 Division of International Epidemiology and Population Studies, Fogarty International Center, National Institutes of Health, Bethesda, MD, USA

## Abstract

*Plasmodium falciparum *malaria is a serious tropical disease that causes more than one million deaths each year, most of them in Africa. It is transmitted by a range of *Anopheles *mosquitoes and the risk of disease varies greatly across the continent. The "entomological inoculation rate" is the commonly-used measure of the intensity of malaria transmission, yet the methods used are currently not standardized, nor do they take the ecological, demographic, and socioeconomic differences across populations into account. To better understand the multiplicity of malaria transmission, this study examines the distribution of transmission intensity across sub-Saharan Africa, reviews the range of methods used, and explores ecological parameters in selected locations. It builds on an extensive geo-referenced database and uses geographical information systems to highlight transmission patterns, knowledge gaps, trends and changes in methodologies over time, and key differences between land use, population density, climate, and the main mosquito species. The aim is to improve the methods of measuring malaria transmission, to help develop the way forward so that we can better assess the impact of the large-scale intervention programmes, and rapid demographic and environmental change taking place across Africa.

## Background

Malaria is the most common, serious mosquito-borne disease in the world [1], yet the tools and methods used to measure the intensity of its transmission are currently not standardized, nor do they take the ecological, demographic, and socioeconomic differences across populations into account [2-7]. This limits the potential for valid spatial-temporal comparisons, and for proper evaluations of the impact of interventions and environmental changes. The recent resurgence in large-scale malaria control programmes, extensive land use changes and the beginnings of significant global climate change make these limitations increasingly important [8,9].

Today, the greatest burden of malaria occurs across sub-Saharan Africa, where *Plasmodium falciparum*, the most severe of the parasite species that infect humans, is estimated to cause approximately 250 million cases and nearly one million deaths each year [1]. Malaria in sub-Saharan Africa is transmitted by a range of *Anopheles *mosquitoes and the risk of infection and disease vary greatly across the continent. The intensity of malaria transmission may be measured several ways, however, the entomological inoculation rate (EIR) is considered a more direct measure of transmission intensity than incidence, prevalence or other traditional epidemiological estimates. EIR is a commonly used metric that estimates the number of bites by infectious mosquitoes per person per unit time [10]. It is the product of the "human biting rate" – the number of bites per person per day by vector mosquitoes – and the fraction of vector mosquitoes that are infectious (the "sporozoite rate") [11].

Understanding the dynamics of malaria transmission in a population is critical; it provides insight into the magnitude of the problem, helps to define when and where the greatest risk occurs and facilitates the development of appropriate control strategies [12-15]. Furthermore, it is important to determine how the level of risk within a population may compare with other (or surrounding) populations – this will help identify key differences and similarities and highlight corresponding risk factors. It is a well-known, but still sparsely-documented fact in sub-Saharan Africa, that villages only a few km apart can have EIRs differing 10× or more, and that such differences can profoundly affect factors such as the prevalence of infection, age incidence and symptomatic presentation of clinical disease, development of immunity, drug use and drug resistance [16-18]. Measuring transmission over longer periods can also help define intra- and inter-annual variability as well as assess the impact of changes within a population such as the introduction of a particular intervention (e.g. indoor residual spraying (IRS) of insecticides and distribution of insecticide-treated bed nets (ITNs)) [19-21], migration, and/or changes in climate and land use patterns (e.g. irrigated agriculture, urbanization) [4-6].

Several recent reviews of EIR data [4-6], have helped to highlight the variable spatial and temporal patterns of malaria transmission, differences between demographic and ecological settings – especially urban and rural populations – and the use of a range of methods to measure transmission across Africa. Importantly, these reviews note the absence of data standardization between studies, raise issues of data quality and information technology, and suggest improvements for future studies.

However, there remains a lack of consensus among the malaria science community and those involved in vector control activities, regarding the best and most efficient way to measure malaria transmission. To address this issue and to better understand the multiplicity of malaria transmission, this current study builds on the recent comprehensive review by Hay *et al *[6]. Specifically, it examines the distribution of annual EIRs across sub-Saharan Africa, reviews the range of methods used, explores ecological parameters in selected locations and makes a series of recommendations regarding the way forward.

## Methods

The analyses in this study are primarily based on the information available from the systematic meta-analysis of annual *P. falciparum *(A*Pf*) EIRs carried out by Hay *et al *[6], which included 233 A*Pf *EIR estimates, from 23 countries across Africa between 1980 and 2004. All references and data may be obtained from the original paper [6]. Additional information on the measurement methods and main *Anopheles *mosquito species were obtained from the geo-referenced database found on Mapping Malaria Risk in Africa (MARA/ARMA) website [22], based on Hay *et al *[4], and supplemented with data from the literature.

First, to examine the distribution of A*Pf *EIRs across sub-Saharan Africa over the 25 year time period, each estimate with a geographic reference i.e. latitude and longitude, was compiled in a database, and mapped using the geographic information system (GIS) software ArcGIS (ESRI 9.2, Redlands, CA). Data were summarized by country, and differences in demography, topography and climate were explored by examining the relationship between A*Pf *EIRs, and population density (persons per km^2^), elevation (metres above sea level) and climate suitability (number of months) using the Gridded Population of the World Version 3 (GPWv3) [23,24], Global 2' Elevation Data, ETOPO2 [25] and Seasonal Climatological Suitability for Malaria Transmission [26] maps respectively; the latter map is based on the number of months during the year when climatological averages meet empirically-derived thresholds of precipitation, temperature and relative humidity, and provides more detailed information than the one developed by Craig *et al *[27]. At each A*Pf *EIR location, the corresponding/underlying data on population density, elevation and climate suitability were extracted in ArcGIS and exported for analysis. All descriptive and statistical analyses were undertaken in Microsoft Excel and SPSS 15.0 (SPSS, Inc, Chicago, IL).

Second, in order to fully understand the range of methods used to measure A*Pf*EIRs, all geo-referenced estimates with information on the human biting rate sampling and sporozoite rate detection techniques (the two main measurement components) were compiled. Data were stratified by the different methods, time and location to determine if there were distinct patterns of usage. Further, the study examined the main measurement methods, it stratified A*Pf*EIRs by land use categories Urban, Peri-urban, Rural 1 (100–250 persons per km^2^) and Rural 2 (<100 persons per km^2^) as defined by Hay *et al *[6], and tabulated differences between main categories of population density, elevation and climate suitability. In addition, data related to the main mosquito species complexes *Anopheles gambiae *and *Anopheles funestus *were summarized.

Third, to explore the relationship between urban and adjacent rural locations, areas with large differences in A*Pf *EIRs that had been measured using the same method, were at similar elevations and within close proximity (≤ 20 km) were examined as case studies. Estimates were plotted against maps and satellite images showing the a) 'Urban-Rural Extent' – a global database of urban extents developed as part of the Global Rural-Urban Mapping Project (GRUMP) [24,28], b) 'Earth at Night (City Lights) 5 km' – NASA (layer) night-time lights view of the Earth [29,30], c) 'Cloud Free Earth 1 km' – NASA (layer) symbolized display presentation of the World Cloud Free image data set [31], d) African Land Cover at 150 m resolution (Earth Satellite Corporation) [32].

## Results

### A*Pf*EIR distributions

**Figure 1:**
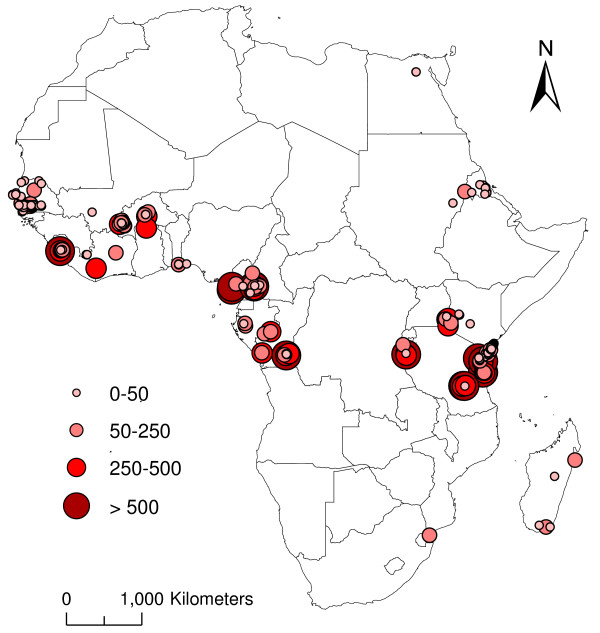
**The magnitude and geographical distribution of annual A*Pf *EIR estimates across Africa between 1980 and 2004**.

In total, A*Pf *EIR estimates were available from 23 (43%) of the 54 African countries (Table 1), with 56% of the measures from four countries alone; Kenya (n = 50), Burkina Faso (n = 30), Tanzania (n = 26) and The Gambia (n = 25). The geographical distribution of all geo-referenced A*Pf *EIR estimates (n = 230) is shown in Figure 1, and highlights the large differences found within and between countries, with values ranging from 0 to 1,030 infective bites per person, per annum. Figure 1 also shows the huge geographic gaps (e.g. Congo) in A*Pf *EIR estimates.

**Table 1:** Summary of A*Pf *EIR estimates by country

**Country**	**N**	**Average A*Pf *EIR**
Benin	6	31.5
Burkina Faso	30	100.6
Burundi	5	251.6
Cameroon	14	184.9
Congo	4	186.6
Congo (D.R)	6	231.0
Cote d'Ivoire	2	314.7
Egypt	2	0.9
Equatorial Guinea	2	814.3
Eritrea	8	14.6
Gabon	6	108.4
Gambia	25	34.8
Ghana	1	418.0
Kenya	50	43.4
Liberia	4	21.9
Madagascar	5	39.5
Mali	1	3.6
Mozambique	1	52.9
Nigeria	1	48.0
Senegal	19	25.3
Sierra Leone	14	155.7
Sudan	1	0.6
Tanzania	26	285.2

**Total**	**233**	**112.2**

**Figure 2:**
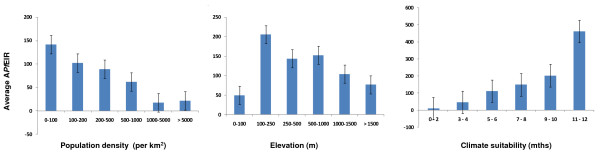
**Average A*Pf*EIR estimates by population density, elevation and climate suitability groupings**. Note. Numbers (n) for population density categories 0–100 (n = 130), 100–200 (n = 26), 200–500 (n = 38), 500–1000 (n = 12), 1000–5000 (n = 13), > 5000 (n = 11); for elevation categories 0–100 (n = 97), 100–250 (n = 38), 250–500 (n = 54), 500–1000 (n = 23) 1000–1500 (n = 13), > 1500 (n = 5) and; for climate suitability categories 0–2 (n = 16) 3–4 (n = 51), 5–6 (n = 89), 7–8 (n = 46), 9–10 (n = 25), 11–12 (n = 3).

The examination of A*Pf *EIRs by population density, elevation and climate suitability indicated that the highest rates occurred in less populated (i.e. rural) places, at sites between 100 m and 1,000 m elevation, and in locations with a higher number of climatically suitable months (Figure 2). For example, average A*Pf *EIRs, were i) 5 times higher in locations with less than 1,000 person per km^2 ^(A*Pf *EIR = 98.7) compared with those with more than 1,000 persons per km^2 ^(19.4); ii) 1.5 to three times higher in locations at 100 to 1,000 m (A*Pf *EIRs = 167) compared with those at lower (49.8) or higher elevations (90.4) and; iii) five times higher in locations with seven or more months (A*Pf *EIRs = 270.5) of climate suitability compared with those with six or less (55.5). Figure 2 also shows that a high proportion of measurements were recorded in populations of low density i.e 0–100 per km^2 ^(n = 130; 57%), in low elevations i.e. 0–100 m (n = 97; 42%) and in locations with five to six months climate suitability (n = 89; 39%).

The relationship between A*Pf*EIRs and each dependent variable was further examined using bivariate correlations, Pearson's correlation coefficient (2 tailed *P *values ≤ 0.05 significance). Due to the large differences in A*Pf*EIRs, population density and elevation estimates, these variables were first transformed to the natural logarithm (log). Analyses indicated a significant negative correlation between A*Pf*EIRs and population density (r = -0.298,*P *≤ 0.01), and significant positive correlation with elevation (r = 0.288, *P *≤ 0.01) and climate suitability (r = 0.456, *P *≤ 0.01).

### A*Pf*EIR measurement methods

In total, 199 study locations reported both the human biting rate sampling and sporozoite rate detection technique. Overall, eleven different methods used to measure A*Pf *EIRs between 1980 and 2004 were identified (Table 2). Human biting rates were most commonly determined using human bait collections (HBC) or pyrethrum spray catches (PSC), however, light traps and window exit traps were also used in some regions and in combination with other methods. Sporozoite rates were primarily determined by the dissection of mosquito salivary glands [3], or by enzyme-linked immuno-sorbent assays (ELISA) [33]. Only one study used the more recently developed polymerase chain reaction (PCR)-based method [34].

**Table 2:** Number of times the different A*Pf*EIR methods were used (at different time intervals) over the 25 year study period

**Sporozoite Detection and Biting Rate Method**	**Year intervals**					
	**1980–84**	**1985–89**	**1990–94**	**1995–99**	**2000–04**	**Row Total**
		
Dissection + HBC	13	19	18	11	-	61
Dissection + PSC	8	-	-	-	-	8
Dissection + Exit Trap	-	2	-	-	-	2
Dissection + ELISA + HBC	-	-	-	1	-	1
ELISA + HBC	-	9	14	13	2	38
ELISA + PSC	-	-	11	31	-	42
ELISA + Light Trap	-	13	10	9	-	32
ELISA + HBC + PSC	-	-	4	-	-	4
ELISA + HBC + PSC + Light Trap	-	-	-	3	-	3
ELISA + HBC +Exit Trap	-	-	5	-	-	5
ELISA + PCR + HBC	-	-	-	3	-	3

**Column total**	**21**	**43**	**62**	**71**	**2**	**199**

Note. Human bait catch = HBC, pyrethrum spray catches = PSC, enzyme-linked immuno-sorbent assays = ELISA, polymerase chain reaction = PCR

**Figure 3:**
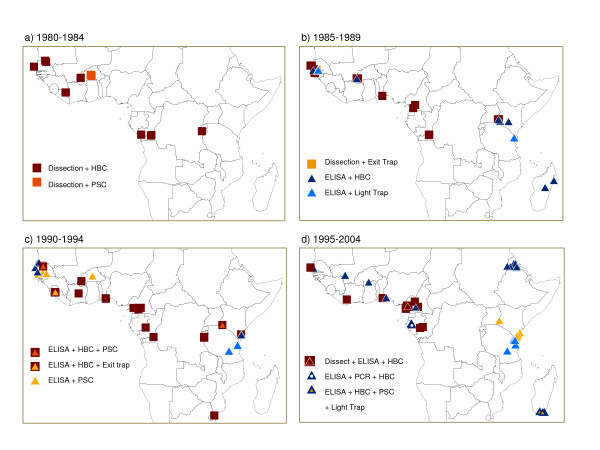
**Geographical distribution of the different methods used to measure malaria transmission at different time intervals between 1980 and 2004**.

**Figure 4:**
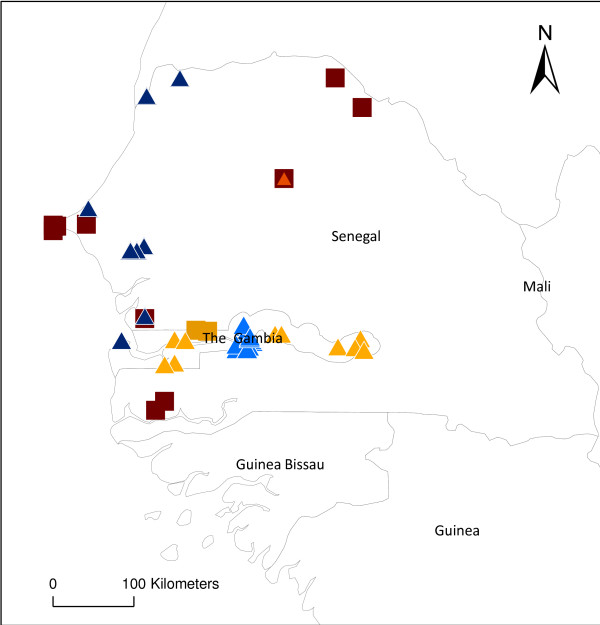
**Close-up of the geographical distribution of the measurement methods (Subset of Figure **3**)**.

Table 2 highlights the overall increasing trend of A*Pf*EIR measurements over time with a total of 21 in 1980–84, and 71 in 1995–99. This table also shows the range of combined methods used, and how their frequencies of use changed over time. New methods joined, but did not entirely replace the old, e.g., as the combinations reported rose from two in the early 1980s to seven in the late 1990s, the use of PSC+ELISA rose from 0% to 44% of estimates, while HBC+dissection dropped from 62% to 15%. There was no distinct geographical pattern in the methods, as illustrated over four different time periods in Figure 3. In some regions, several different methods had been used to measure A*Pf *EIRs (Figure 4).

**Figure 5:**
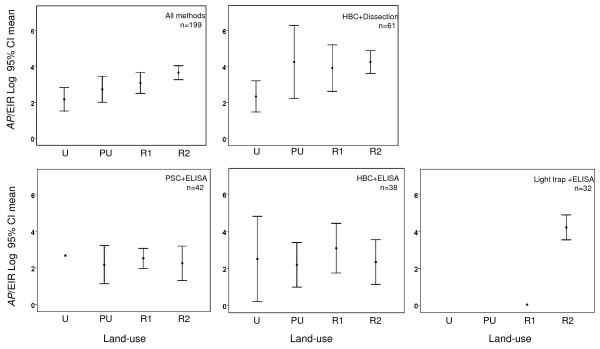
**Graphs of A*Pf*EIR estimates by four main measurement methods and land use categories**.

Overall, the HBC+dissection (n= 61), HBC+ ELISA (n = 38), PSC+ ELISA (n = 42) and light trap+ ELISA (n = 32) combinations were the most frequently used methods, and comparisons of mean A*Pf *EIRs by land use categories showed that these four different methods exhibited different patterns (Figure 5). Significant differences were found between Urban and Rural 2 locations when measured using all methods (in accordance with Hay *et al *[6]), or HBC+dissection, however, these trends were not evident when other methods were used. Further, the light trap+ELISA method was found to have been used only in rural locations, predominantly Rural 2.

The trends shown in Figure 2 persisted across the different measurement methods, and are summarized in Table 3. Due to the relatively small numbers in each measurement group, the dependent variables were examined by two main categories to highlight overall differences in average A*Pf*EIRs. Means were compared using the Mann-Whitney *U *test with Bonferroni correction for multiple comparisons (adjusted *P *values ≤0.004 significance). Overall, locations with low population density (≤ 500 persons per km^2^; A*Pf *EIR = 105.4) and more months of climate suitability (>5 months; A*Pf *EIR = 132.8) had significantly higher rates than those with high population density (>500 persons per km^2^; A*Pf *EIR = 73.9), and fewer months (0–5 mths; A*Pf *EIR = 77.5). Overall, there was no significant difference between locations with lower (≤ 500; A*Pf *EIR = 101.1) and higher (>500; A*Pf *EIR = 137.0) elevation.

**Table 3:** Average A*Pf*EIRs by population density, elevation and climate suitability using different measurement methods

	**HBC + Dissection**	**HBC + ELISA**	**PSC + ELISA**	**Light trap +ELISA**
**Population density**				
≤ 500 person/km^2^	198.6 (45)*	96.4 (32)	21.2 (38)	180.7 (32)
>500 person/km^2^	37.0 (16)	28.7 (6)	20.0 (4)	(0)
**Elevation**				
≤ 500 m	147.7 (41)	70.7 (26)	20.5 (41)	206.4 (28)
>500 m	173.9 (20)	118.1 (12)	46.7 (1)	0.9 (4)
**Climate Suitability**				
0–5 mths	62.4 (19)*	32.0 (30)*	20.5 (41)	95.3 (15)
>5 mths	198.7 (42)	287.1 (8)	46.7 (1)	256.0 (17)

Note: * denotes statistically significant difference between mean A*Pf*EIR estimates in the different population density, elevation and climate suitability groups.

**Figure 6:**
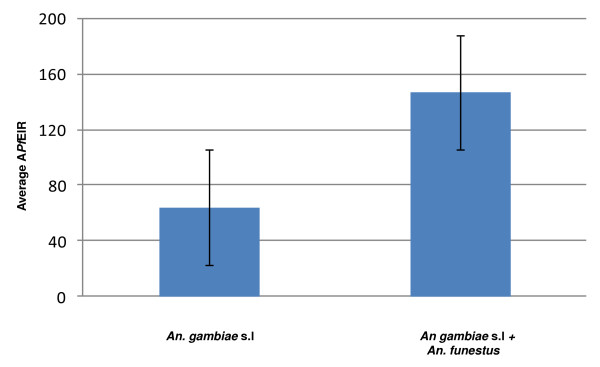
**Comparison of average A*Pf*EIR estimates by two different mosquito species groupings**.

There was great variability in the reporting of the two main mosquito species (complexes), *An. gambiae *s.l and *An. funestus*, and the degree to which each contributed to malaria transmission. Therefore, these A*Pf*EIR comparisons were limited, and examined simply in terms of i) *An. gambiae *s.l – sole species present or identified as responsible for 100% of transmission, and ii) *An. gambiae *+ *An. funestus *– both species present and/or found to be partially responsible for transmission in varying proportions. In total, 187 locations reported information on mosquito species, and overall, average A*Pf *EIRs were found to be more than twice as high in locations where both *An. gambiae *+ *An. funestus *were present (A*Pf *EIR = 147; n = 110), than in locations, which only comprised *An. gambiae *s.l. (A*Pf *EIR = 64; n = 77) (Figure 6). Further comparison of the means (Mann-Whitney *U *test with Bonferroni corrected *P *values ≤ 0.004), of the two species groups by measurement methods, land use, population density, elevation and climate suitability categories showed similar trends, with the greatest differences and highest average A*Pf *EIRs found in locations of low population densities i.e. rural, those with a high number of climatically suitable months i.e. 6–12, and where the HBC+dissection method was used (Table 4).

**Table 4:** Comparisons of average A*Pf*EIRs in the presence and absence of *An. gambiae *and *An. funestus *by measurement methods, land use, population density, elevation and climate suitability

	***An. gambiae *s.l (n = 77)**	***An. gambiae + An. funestus *(n = 110)**	**Signif.**
**Measurement Method**			
HBC + Dissection	64.3 (28)	266.45 (29)	*
HBC + ELISA	86.7 (12)	100.7 (22)	
PSC + ELISA	2.6 (15)	31.4 (27)	*
Light trap + ELISA	103.5 (13)	225.36 (18)	
**Land use**			
Urban	25.3 (12)	27.2 (6)	
Peri-urban	105.6 (6)	86.2 (22)	
Rural 1	90.5 (10)	115.7 (31)	
Rural 2	63.0 (49)	206.2 (51)	*
**Population density**			
≤500 person/km2	73.2 (63)	159.1 (96)	*
>500 person/km2	22.5 (14)	63.1 (14)	
**Elevation**			
≤500 m	67.9 (64)	134.2 (86)	*
>500 m	44.6 (13)	192.6 (24)	
**Climate Suitability**			
0–5 mths	24.6 (49)	68.0 (59)	*
>5 mths	132.9 (28)	238.3 (51)	

Note: * denotes statistically significant difference between mean A*Pf*EIR estimates in different mosquito species groupings

Additionally, there was great variability within each method, particularly for human-biting-rate sampling techniques. Most studies provided little or no explanation regarding the rationale behind the choice of locations, houses, or trap placements, nor information on the human collectors, time of day, or frequency of mosquito collection. It was also found that PSC-based studies did not routinely take the mosquito species composition into account, an omission, which may be important in locations where vectors often rest outdoors after feeding.

### Case studies

#### Burkina Faso (Figure 7, maps a-d)

**Figure 7:**
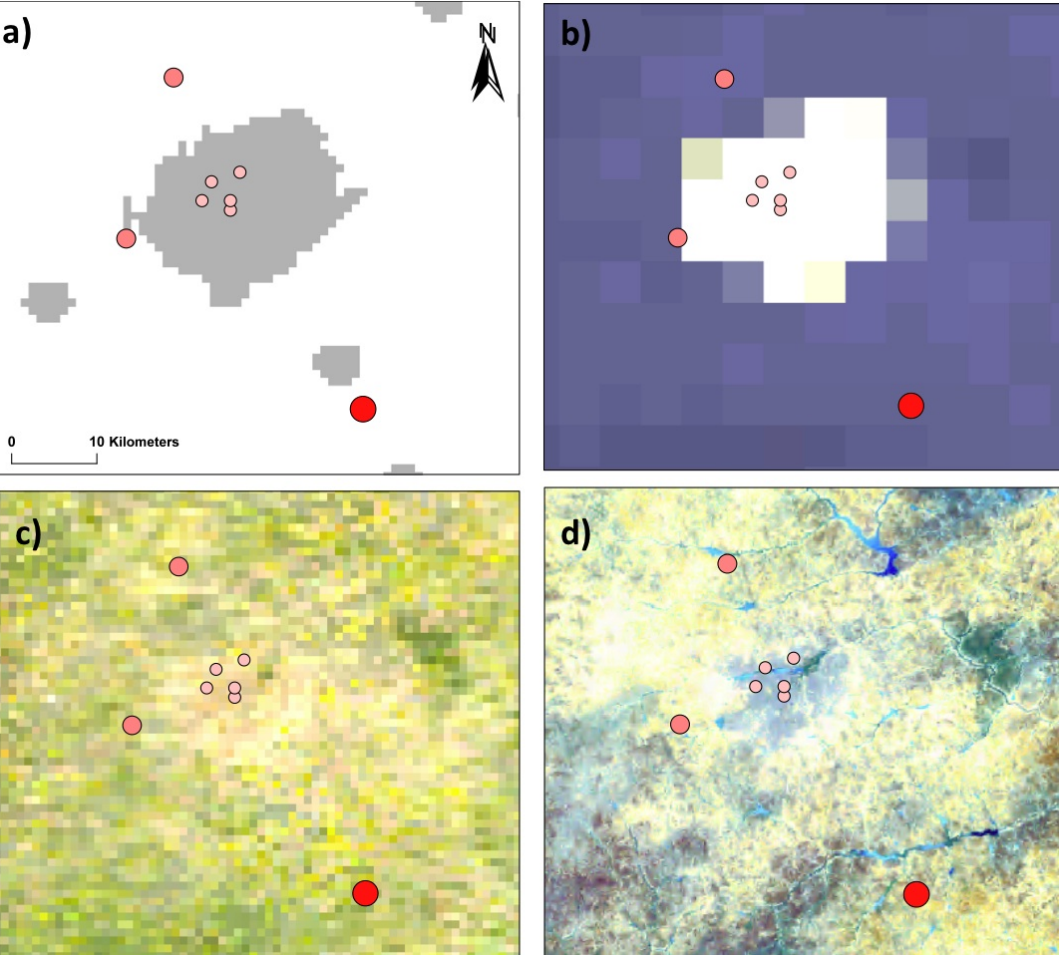
**Burkina Faso case study**. a) Urban-Rural Extent b) Earth at Night (City Lights) 5 km c) Cloud Free Earth 1 km  d) African Land Cover at 150 m.

In 1984, a longitudinal malaria survey was carried out in the capital city, Ouagadougou, and three nearby villages (three months climate suitability) [35]. A*Pf *EIRs were determined from PSC and dissections. The main mosquito vectors were *An. gambiae *s.l and *An. funestus*. In urban Ouagadougou (Pop. density ~1,560 persons per km^2^), A*Pf *EIRs ranged from 0 to 7.7, which were significantly lower than those recorded in a rural village (with irrigation) 10 kms west (A*Pf *EIR = 82; Pop. density~1136)), a rural village 15 kms north (A*Pf *EIR = 113; Pop. density~488) and a rural village 30 kms south (A*Pf *EIR = 442; Pop. density~67) from the city centre (maps a-d). Thus, transmission appeared to increase with distance from the urban area with an estimated 2.5 to 5 rise in A*Pf *EIRs per kilometre. The maps further highlight the reduced risk of malaria in urban areas (map a – grey shading) and where city lights prevail (map b).

#### Benin (Figure 8, maps a-d)

**Figure 8:**
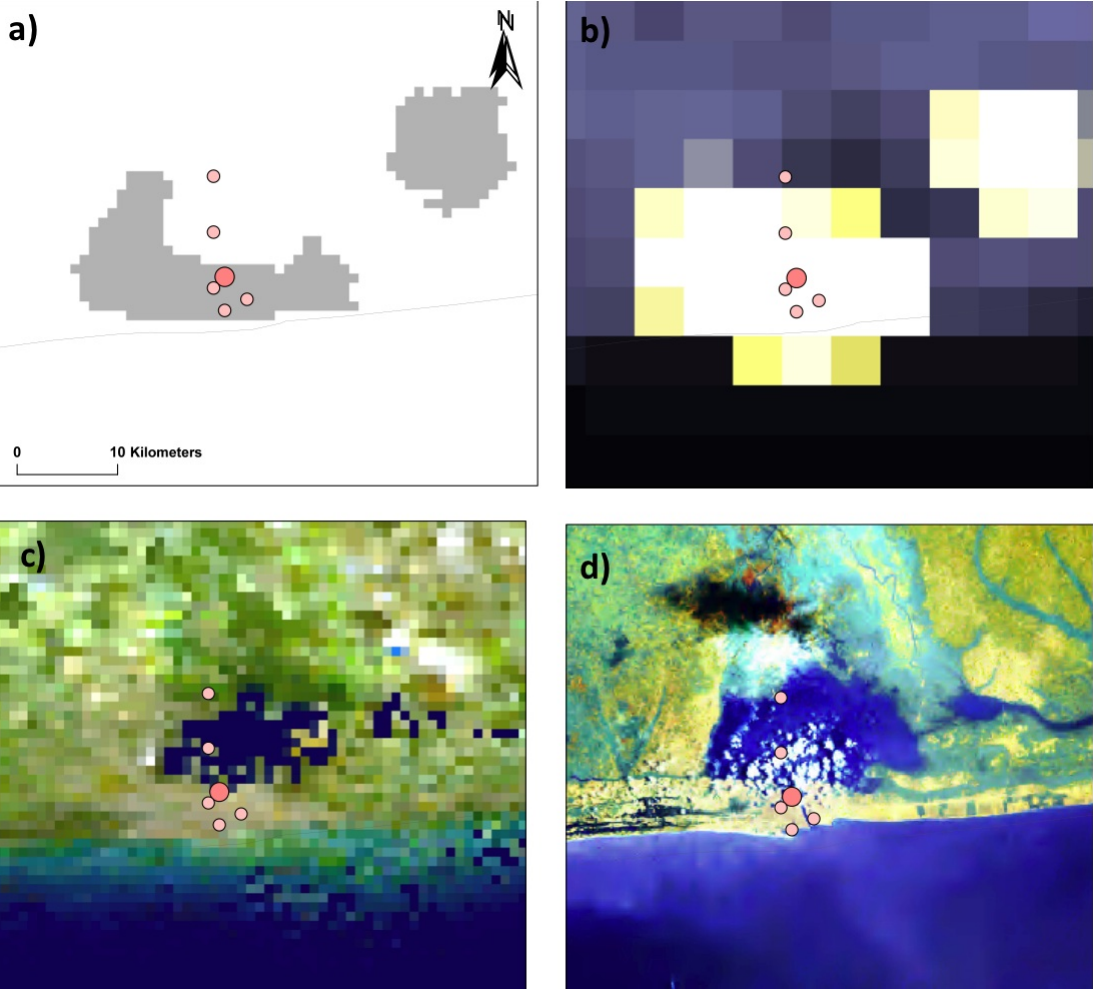
**Benin case study**. a) Urban-Rural Extent b) Earth at Night (City Lights) 5 km c) Cloud Free Earth 1 km d) African Land Cover at 150 m.

Three studies were undertaken in the coastal town of Cotonou and a village on Nokoué Lake between 1987 and 1995 (eight months climate suitability) [36-38]. A*Pf *EIRs were determined from HBC and dissections. The main mosquito vectors were *An. gambiae *s.s and *Anopheles melas*; the latter being the most abundant in the lagoon areas, and, although an aggressive biter, considered a poor malaria vector. This may account for the low transmission found in the traditional village by the lake where A*Pf *EIRs were 11 (Pop. density ~744 persons per km^2^), which was, on average, four times lower than that found in urban Cotonou where A*Pf *EIRs ranged between 33 and 58 (Pop. density ~3035–12,341), some 10 kms across the lake (maps a-d). Freshwater *An. gambiae *s.s is considered to be an important vector in urban Cotonou, despite the narrow strip of land between the Atlantic Ocean and Nokoué Lake, on which the town sits.

#### Republics of Congo (Figure 9, maps a-d)

**Figure 9:**
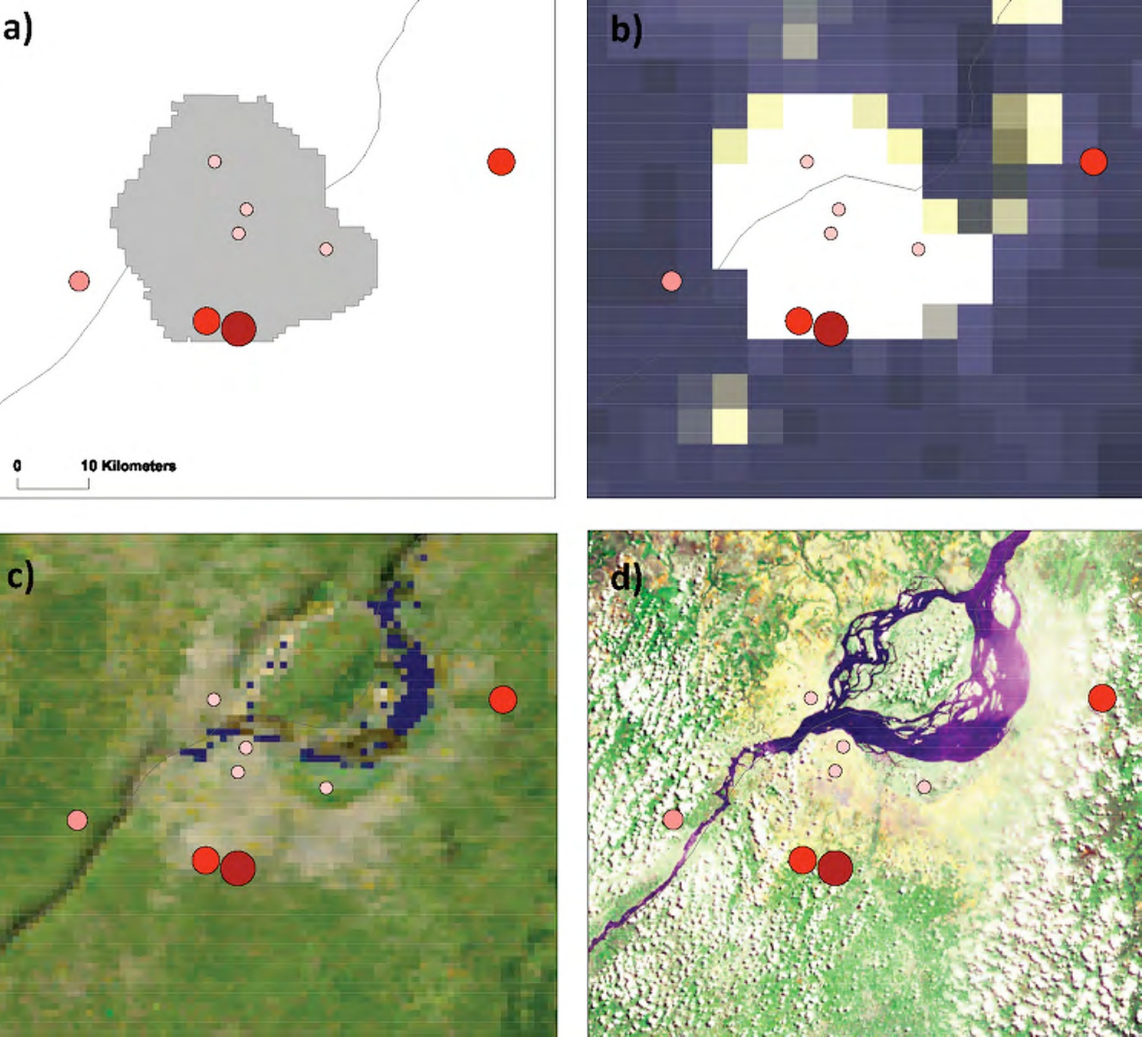
**Republics of Congo case study**. a) Urban-Rural Extent b) Earth at Night (City Lights) 5 km c) Cloud Free Earth 1 km d) African Land Cover at 150 m.

In the capital cities and surrounding rural areas of the Republic of Congo (Brazzaville) and Democratic Republic of Congo (Kinshasa), entomological studies were carried out between 1981 and 1984, and 1989 and 1991, respectively (eight months climate suitability) [39-42]. A*Pf *EIRs were determined from HBC and dissections. The main vector was *An. gambiae *s.l. Both cities had similar patterns with lower transmission (A*Pf *EIR; 2.9, 22.5) than the adjoining peri-urban (A*Pf *EIR; 31) and rural areas (A*Pf *EIR; 24, 246, 620), but with less strongly inverse relationships with population density patterns (Urban/peri-urban Pop. density~208–7954 vs. rural ~186–7954). The maps indicate that the urban area of Kinshasa (maps a, b) has grown since the study and now includes areas previously classified as peri-urban and rural. This suggests that the ecology and risk of malaria may have changed in these locations in recent years.

## Conclusion

This review shows the geographical distribution of A*Pf *EIR estimates across sub-Saharan Africa, the region at greatest malaria risk in the world [1], and highlights the vast gaps in knowledge on the transmission of this devastating disease. The fact that only half the countries of sub-Saharan Africa have data available on transmission intensities is of great concern. This dearth of fundamental data raises questions about how current large scale vector control, malaria elimination and eradication programmes currently underway across the continent can develop realistic plans to achieve their goals [43-49]. It emphasizes the need for systematic sampling across a wider geographical area, to include a more diverse range of demographic and ecological settings.

The highest number of A*Pf *EIR estimates were taken in rural populations, in particular in locations with <100 person per km^2^(n = 130). Very few measures were taken in urban areas where the population density was high i.e. > 1,000 per km^2^. Hence, there is a pressing need to know more about urban population transmission dynamics, given the rapid urbanization currently taking place across the continent [50]. It is predicted that more than 60% of the population in sub-Saharan Africa will be urban dwelling by 2020. The examination of modelled population density to define differing demographic trends, suggests that they may be preferable to the urban-rural categories defined by Hay *et al *[6], which are potentially subjective e.g. urban areas can vary greatly in population density as elucidated in the three case studies.

Importantly, there was also great variation in the number of estimates taken in areas of different elevations and months of climate suitability. Approximately 40% of the A*Pf *EIR estimates were taken at elevations of <100 m, and these were, on average, significantly lower than all those taken at higher elevations. This apparent lower risk at lower elevations may be associated with the measurements taken in coastal locations where mosquito species such as *An. melas *and *Anopheles merus *prevail, but are considered to be poor vectors of malaria [36-38,51-53]. It may also be related to urban populations, which are usually located at lower elevations and have better access to anti-malarial drugs [54]. More importantly, nearly 80% of A*Pf *EIR estimates were taken in areas where climate suitability [26] was six months or less. This results in estimates from short transmission periods being extrapolated to average annual rates, thereby introducing inaccuracies. This points to whether A*Pf *EIR estimates would be better presented as monthly measures over a year, highlighting the seasonality, as well as local demographic and ecological factors such as interventions, community wealth and land use e.g. irrigation, which could potentially shorten or prolong the transmission season(s).

Bivariate correlations indicated that population density, elevation and climate were all significantly related to A*Pf *EIRs and important factors influencing the risk of transmission. Whilst these data and analyses are crude, in the absence of ground-truth data they provide some useful insights into potentially important associations, which can be followed up in more detail and depth. This work also highlights the advantages of using state of the art GIS tools and remote sensing (RS) technologies [55], especially with changes in population and climate becoming increasingly important to monitor in under-resourced regions of the world such as sub-Saharan Africa [8,9].

The work on the methods used to measure A*Pf *EIRs is the most comprehensive review available. It highlights the overall increasing trend over time with a total of 21 measurements recorded in 1980–84, compared with 71 in 1995–99. Of note, however, few transmission studies have been undertaken in recent years. The reason for this lack of work is unclear, but may be related to the limited infrastructure and overall lack of financial resources, trained staff, vector ecologists and medical entomologists on the ground [56]. This may also explain why the methods have varied so much over time, with the more labour intensive and specialized method of HBC to catch blood-fed mosquitoes and the sporozoite dissection technique, being replaced with different combinations of PSC, light traps, ELISA and PCR, which are quicker and simpler. This shift in methodological approaches is probably due to both a lack of human and financial resources, and ethical reasons related to the increasing prevalence of drug resistance across the continent [57].

The use of so many different methods has reduced the ability to compare malaria transmission dynamics within and between populations over time. Although there have been attempts to calibrate and understand the relationship between the different HBC [58-64] and sporozoite detection [65-72] methods, the advantages and disadvantages of each method have not been thoroughly examined, and it is still not known how they compare over long periods of time in different settings. This highlights the need for a simpler and more standardized method for measuring A*Pf* EIRs, a point previously emphasized by Service [3] and Hay *et al *[4]. Alternative approaches such as the use of immunological tools (in combination with entomology) also need to be considered, as they have the potential to evaluate the medium- and long-term trends of transmission, and determine the influence of *Anopheles *vectors species on the regulation of antibody responses to *P. falciparum *[73-75]. This is critical for future studies to better understand the complexities of transmission and the impact of the changes occurring across Africa in terms of urbanization, climate change and large-scale intervention and control programmes involving the mass distribution of ITNs and extensive IRS [43-49].

This current study shows that there are clear and significant differences between urban and rural populations, when A*Pf*EIRs are measured using all methods (in accordance with Hay *et al *[6]), or HBC and dissection. However, these trends were not evident when other method combinations were used, nor when other demographic and ecological factors were stratified by different methods. The reasons may be that there is great variability within each method, and transmission heterogeneity within each location [76-79], both important factors that potentially were not taken into account. Most studies provided little or no explanation regarding mosquito distribution patterns, the rationale behind the choice of locations, houses, or trap placements, nor information on the human collectors, time of day, or frequency of mosquito collections. Further, it was found that PSC-based studies [80-83] did not routinely adjust for the different feeding and resting patterns of the mosquito species, an omission, which may be important in locations where vectors often rest outdoors after feeding e.g. *Anopheles arabiensis *[84].

Although they are difficult to measure, phenotypic variables like exophily, and genotypic variation in vector competence are also important considerations. Our simple comparisons between *An. gambiae *s.l and *An. funestus *presence show how crucial it is to take the *Anopheles *species characteristics and ecological niches into account. Malaria transmission appears to vary greatly by mosquitoes species, depending upon the land use, population density, elevation and climatic parameters [6]. Overall, A*Pf *EIRs were twice as high in locations where both *An. gambiae *s.l and *An. funestus *were present, compared with locations which only comprised *An. gambiae *s.l. The greatest differences and highest A*Pf* EIRs occurred in locations of low population densities i.e. rural, those at elevations of 500 to 1,000 m and where periods of suitable climate exceeded six months.

These preliminary findings indicate that *An. funestus *is an important, yet potentially underappreciated, vector contributing to high levels of malaria transmission across sub-Saharan Africa. This may help to explain why some populations in close proximity have vastly different A*Pf* EIRs, as exemplified in Senegal, in the village of Dielmo where *An. funestus *was abundant and transmission 10× higher than the village Ndiope (5 km away), where *An. funestus *was rare [17,18]. Furthermore, *An. funestus *is seldom found in urban areas, and, where rainfall is confined to a single season each year, is typically most abundant at the end of that season and beginning of the dry season that follows [17,75,76,85-89]. Hence, in line with the other factors considered here, one important role of *An. funestus *is in extending the transmission season in rural areas. More specific research on this vector species is critical – it is a notoriously difficult vector to find in the field, catch and colonise.

The three case studies presented in this paper provide further insight into the ecological factors influencing the diverse mosquito distributions and *P. falciparum *malaria epidemiology across sub-Saharan Africa. Of note, and perhaps of most concern is the great variation in risk that occurs within relatively small geographical areas, especially in and around urban areas. The implications for a growing urban Africa are unclear. Will urbanization decrease the risk of malaria? Will the mosquito vectors commonly found in peri-urban or adjacent rural areas adapt to urban environments and increase the risk of malaria? How will we measure this? An improved approach to measurement will have numerous ramifications, some perhaps not widely anticipated. For instance differences in transmission intensity, and corresponding immunity, might help to explain circumstances in which frequencies of drug failure differ, far more than frequencies of drug-resistance markers, between urban and surrounding rural areas [90]. This review highlights the complexity and multiplicity of malaria transmission, and serves as a foundation from which to move forward, to develop sensible and realistic methods for measuring malaria transmission in Africa today and for the future.

## Competing interests

The authors declare that they have no competing interests.

## Authors' contributions

LKH designed the study, identified data sources, carried out the data analysis and wrote the first draft of the manuscript. EM conceived the idea for the article and contributed significantly to the formatting and writing of the manuscript.
